# Complete mitochondrial genomes of representatives of two endemic sculpin families (Perciformes: Cottoidei) from Baikal – the world’s largest and deepest lake

**DOI:** 10.1080/23802359.2021.1989330

**Published:** 2021-10-14

**Authors:** Nikolai Mugue, Anna Barmintseva, Anna Etingova, Sergei Didorenko, Maria Selifanova, Lubov Mugue, Alexei Popov, Andrei Bulakhov, Aleksandr Kupchinskiy

**Affiliations:** a Russian Institute for Fishery and Oceanography (VNIRO), Moscow, Russia; b Koltzov Institute of Developmental Biology RAS, Moscow Russia; c The Baikal Museum at the ISC SB RAS, Listvyanka, Russia; d Moscow State University, GSP-1, Leninskie Gory, Moscow, Russia; e Skolkovo Institute of Science and Technology, Skolkovo, Russia

**Keywords:** *Cottocomephorus inermis*, *Cottocomephorus grewingkii*, *Paracottus knerii*, *Procottus major*, Lake Baikal

## Abstract

In this study, five new mitogenomes from four endemic Lake Baikal sculpins were determined: *Cottocomephorus grewingkii* (Dybowski, 1874) (GB#MW732165), *Cottocomephorus inermis* (Yakovlev, 1890) (GB#MW732163), and *Paracottus knerii* (Dybowski, 1874) (GB#MW732164) (Family Cottocomephoridae – Bighead sculpins), and from two specimens of *Procottus major* Taliev, 1949 (GB##MW732166, MW732167) from Family Abyssocottidae (Deep-water sculpins). Together with recently published mitogenomes of Baikal Oilfishes (Sandel et al. 2017), the first mitogenome-based phylogenetic tree for all three endemic Baikal sculpin families is presented. Complete mitogenome phylogeny supports the monophyletic origin of the lake Baikal sculpins species flock, but does not support the monophyly of the family Cottocomephoridae (Bighead sculpins).

Lake Baikal is an extraordinary hotspot of biodiversity, located in temperate latitudes. With more than 1500 endemic species, Lake Baikal is an ideal natural laboratory to study speciation and adaptation to extremely contrasting environmental conditions. Cold and oligotrophic water, high oxygenation from the surface to the very bottom (1650 m), long and relatively stable paleographic history of the lake resulted in flourish the very limited number of endemic taxonomic groups. Lake Baikal sculpins species flock is an outstanding example of adaptive radiation in temperate latitudes. Having a monophyletic origin, an estimated age of less than two million years (Kontula et al. 2003), and the closely related species of the genus *Cottus*, inhabiting rivers and lakes of the Palearctic, this amazing group of fishes has formed a striking endemic complex which accounts 35 species described to date assigned to three families (Taliev 1955; Sideleva 2003). These fish inhabit a variety of bottom substrates from the surface to over 1.6 km depth, and some species have adopted the pelagic zone at colossal depths. For decades Lake Baikal fauna attracts evolutionary biologists throughout the world, however, sculpins remain a largely underexplored group.

From RNA-Seq study, aimed to assess the Lake Baikal sculpin adaptive radiation, we assembled five mitochondrial genomes of four species from two endemic families: *Cottocomephorus grewingkii* (GB#MW732165, Voucher BK19-6), *Cottocomephorus inermis* (GB#MW732163, Voucher BK19-8), and *Paracottus knerii* (GB#MW732164, voucher#19-7) from Family Cottocomephoridae (Bighead sculpins) and two specimens of *Procottus major* (GB#MW732166, voucher#BK19-13 and MW732167, voucher#BK19-13) from Family Abyssocottidae (Deep-water sculpins).

All sculpin specimens for this study were caught in February–March 2019 near the Baikal Museum at Listvyanka village (51.850°N, 104.833°E, Irkutsk District, Russia) by various fishing gears. Gill and liver tissue were collected and stored in RNA-Later solution for further study, and formalin-fixed specimens with voucher numbers assigned are deposited at VNIRO collection (www.vniro.ru, e-mail mugue@vniro.ru).

Total RNA extraction was carried out by PureLink® RNAMiniKit (Invitrogen 12183018 A) with on-column Dnase treatment (PureLink® DNaseSet, Ambion). Barcoded cDNA libraries were prepared with TruSeq® Stranded mRNA LT Kit according to the manufacturer’s protocol. Illumina HiSeq4000 sequencing was conducted at Skolkovo genomic center (Moscow, Russia). Reads were trimmed with Trimmomatic, mapped on *C. baicalensis* mitochondrial genome (NC036148) with SeqMan NGen 12.0.1 (DNASTAR Inc), and each assembly was verified by visual inspection in SeqMan with special attention to protein-coding gene boundaries to trim poly-A tails derived from mRNAs. Average coverage ranged from 3–5× at the control region to over 15,000× (COX1, COX2, COX3, CYTB). Additionally, complete control regions for each species were PCR -amplified from genomic DNA and Sanger-sequenced with primers CottusDL_F (CCTACCCCTAACTCCCAAAGC) and CottusDL_R (TGCTTGCGGGACTTTCTAGG) to verify assembled mitogenomes.

Sequenced genomes have typical for teleosts mitogenome organization with length varies from 16,506 bp to 16,590 bp. The observed length difference is mostly due to variable poly-C stretch found between tRNA-Asp and COX2 gene. Interestingly, a 40 bp repeat (3.2 copies) was found within the control region in *C. grewingkii*, but not in closely related *C. inermis* and other species sequenced.

In the whole genome phylogenetic analysis, we included sequences of Big Baikal Oilfish *Comephorus baicalensis* (Pallas, 1776) and Little Baikal Oilfish *Comephorus dybowskii* (Korotneff, 1904) complete mitochondrial genomes (Sandel et al. 2017) from Baikal sculpin family Comephoridae, and 12 species of genus Cottus as an outgroup. MEGA X (Kumar et al. 2018) was used for multiple alignment and maximum-likelihood phylogenetic analysis (Figure 1). Minimum evolution and neighbor-joining trees resulted in the same tree topology as the maximum-likelihood tree. *Paracottus knerii* do not form monophyletic clade with other species from family Cottocomephoridae, which indicates that further taxonomic revision of this enigmatic group is on demand.

**Figure 1.**
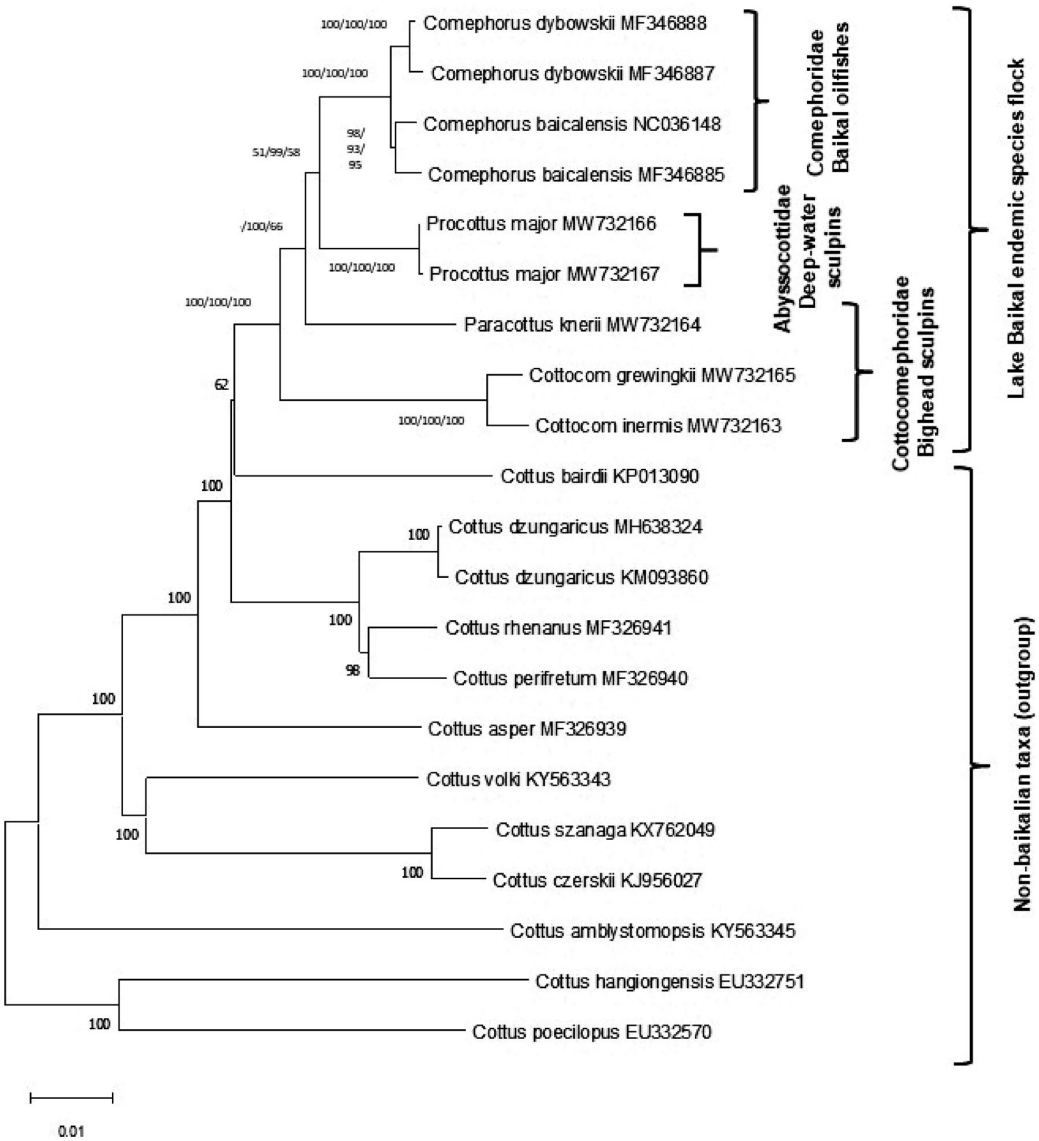
Phylogenetic tree of Baikal sculpins from three endemic families and 12 closely related palearctic *Cottus* species, constructed using the maximum likelihood (GTR + G + I) method based on complete mitogenomes. Bootstrap support for ML/ME/MP is indicated for Baikalian taxa. New sequences are MW732163-7.

## Data Availability

The data that support the findings of this study are openly available in GenBank at [https://www.ncbi.nlm.nih.gov/genbank/], reference numberы MW732163- MW732167.
